# Izumo meets Juno: Preventing polyspermy in fertilization

**DOI:** 10.4161/cc.29461

**Published:** 2014-06-06

**Authors:** Enrica Bianchi, Gavin J Wright

**Affiliations:** Cell Surface Signalling Laboratory; Wellcome Trust Sanger Institute; Cambridge, UK

**Keywords:** Juno, Izumo1, fertilization, polyspermy block

Sexual reproduction ensures the continued propagation of almost all eukaryotic species. Two morphologically very different types of cell, which develop exclusively in either the male or female, perform the role of creating a new and genetically distinct organism. In mammals, these 2 haploid cells, the sperm and the egg, meet in the female reproductive tract, interact, and finally fuse to become a single, diploid cell. Since the development of in vitro fertilization, the cell biology of this process has been well described, but the molecular details, and in particular the receptor proteins involved in sperm–egg recognition, have remained remarkably elusive. A breakthrough was made in 2005 when Masaru Okabe’s group identified a protein displayed on the surface of acrosome-reacted sperm and showed that sperm lacking this receptor were unable to fuse with normal eggs; they named this protein “Izumo” after a Japanese marriage shrine.^1^ Although it seemed likely that Izumo had a partner on the surface of the egg, it remained (as was sometimes said in jest) a bachelor for 9 years. Largely, this was because eggs are a rare cell type, thereby limiting material available for experiments, and that extracellular receptor–ligand interactions are typified by their transient nature, making binding partners challenging to detect.^2^ Using techniques designed to detect these fleeting interactions, we recently identified the egg binding partner for Izumo1 on the egg as folate receptor 4, a protein named by sequence homology to folate receptors, but because we showed that it was unable to bind folate, we suggested renaming it “Juno” after the Roman goddess of marriage and fertility. We further demonstrated that Juno-deficient eggs could not fuse with normal acrosome-reacted sperm, providing evidence that the interaction between Izumo1 and Juno was essential for mammalian fertilization.^3^

One feature shared by all eggs is that they must fuse with one—and only one—sperm. Eggs that fuse with more than a single sperm contain an excessive amount of genetic material contributed by the male and are said to be polyspermic, resulting in the formation of a nonviable embryo. While it is the egg membrane that, once fertilized, shuts down its receptivity to additional sperm, remarkably, different organisms have evolved distinct mechanisms that are appropriate for their reproductive strategies. For example, in broadcast-spawning aquatic animals such as sea urchins and amphibians, millions of sperm are released in close proximity to eggs, requiring a very fast block to polyspermy, which is achieved through the rapid (a few seconds) depolarization of the oolemma, thereby making it almost immediately unreceptive to additional sperm.^4^ By contrast, although male mammals also release hundreds of millions of sperm, the female mammalian reproductive system seems designed to create a stringent selection system, so that only a few hundred sperm actually reach the egg. Despite the restriction in the number of sperm reaching the egg, the oolemma of mammalian eggs also becomes unreceptive to additional sperm after fertilization, a phenomenon first described 60 y ago in rabbits, where unfused sperm were observed in the perivitelline space of recently fertilized eggs.^5^ Further studies in other mammals such as mice showed that this membrane block to polyspermy was much slower than in aquatic organisms, taking around 40 min for eggs to become unreceptive. It was soon established that this block to polyspermy did not require membrane depolarization, but the exact mechanism remained a long-standing mystery. With this in mind, we observed that Juno, which is highly expressed on unfertilized eggs, became undetectable within 30–40 min after fertilization, in close agreement with the timing of the membrane block to polyspermy. Using immunogold electron microscopy, we could show that Juno was shed from the oolemma and redistributed within a field of vesicles confined within the perivitelline space. We believe that these Juno-displaying vesicles could act as rapid sperm-blocking agents, binding to and neutralizing incoming acrosome-reacted sperm, thereby reducing the effective timing of the membrane block, since 40 min may seem like a long time for eggs to remain susceptible to subsequent fatal sperm fusions (Fig. 1). Nevertheless, this mechanism for preventing polyspermy is not flawless, since the incidence of polyspermy in mammals is between 1 and 2%^6^ and around 7% in human in vitro fertilization.^7^

**Figure:**
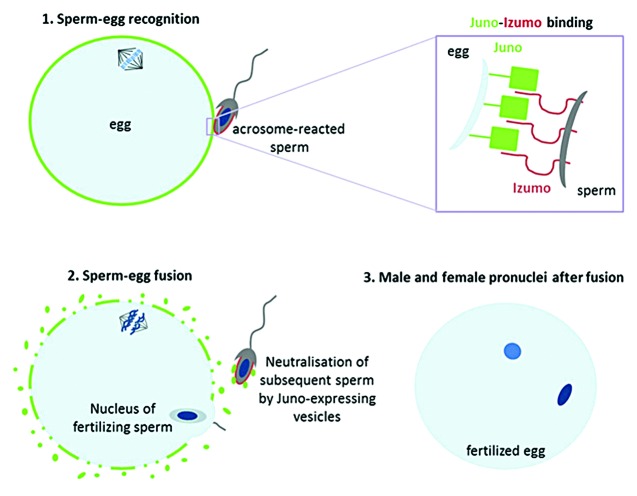
**Figure 1.** Juno–Izumo binding is essential for fertilization and may contribute to the membrane block to polyspermy. Izumo (red) is displayed on the surface of acrosome-reacted sperm and interacts with Juno (green) on the surface of ovulated eggs. Following fertilization, Juno is rapidly shed from the egg membrane and redistributed as vesicles, which may bind and rapidly neutralize subsequent incoming acrosome-reacted sperm, thereby reducing the possibility of creating nonviable polyploid embryos.

While these findings may eventually be used to improve assisted fertility treatments, the essential requirement for the Izumo1–Juno interaction could provide novel opportunities for the development of non-hormonal and/or more flexible contraceptives, something that may be important given the rapid expansion of the human population on a planet of finite resources. Beyond these applications, the identification of the molecules that must have interacted when our father’s sperm and mother’s egg first met at the moment we were conceived solves an intriguing question in the biology of fertilization.

## Notes

BianchiE Nature 2014 10.1038/nature13203

## References

[1] Inoue N (2005). Nature.

[2] Wright GJ (2009). Mol Biosyst.

[3] Bianchi E (2014). Nature.

[4] Jaffe LA (1976). Nature.

[5] Austin CR (1953). Aust J Biol Sci.

[6] Gardner AJ (2006). Reprod Fertil Dev.

[7] Ho PC (1994). Int J Fertil Menop S.

